# A Pediatric Tumor Found Frequently in the Adult Population: A Case of Anaplastic Astroblastoma in an Elderly Patient and Review of the Literature

**DOI:** 10.1155/2017/1607915

**Published:** 2017-01-23

**Authors:** Christopher Payne, Ali Batouli, Kristen Stabingas, Dunbar Alcindor, Khaled Abdel Aziz, Cunfeng Pu, Elizabeth Tyler-Kabara, Robert Williams, Alexander Yu

**Affiliations:** ^1^Department of Neurosurgery, Allegheny General Hospital, Pittsburgh, PA, USA; ^2^Department of Radiology, Allegheny General Hospital, Pittsburgh, PA, USA; ^3^Department of Pathology, Allegheny General Hospital, Pittsburgh, PA, USA; ^4^Department of Neurological Surgery, Children's Hospital of Pittsburgh, University of Pittsburgh, Pittsburgh, PA, USA

## Abstract

Astroblastomas are rare, potentially curable primary brain tumors which can be difficult to diagnose. We present the case of astroblastoma in a 73-year-old male, an atypical age for this tumor, more classically found in pediatric and young adult populations. Through our case and review of the literature, we note that this tumor is frequently reported in adult populations and the presentation of this tumor in the elderly is well described. This tumor is an important consideration in the differential diagnosis when managing both pediatric and adult patients of any age who present with the imaging findings characteristic of this rare tumor.

## 1. Introduction

Astroblastomas are uncommon tumors of neuroepithelial origin first described by Bailey and Cushing in 1926 [1]. These tumors are found in the cerebral hemispheres, most commonly seen in children and young adults, with a reported incidence of 0.45–2.8% [2]. A bimodal distribution of cases has been reported with peak prevalence between 5 and 10 years of age and 21 and 30 years [3]. Astroblastomas present with signs of increased intracranial pressure and currently do not have unified diagnostic criteria [4, 5]. Furthermore, they have similar radiologic and histopathologic features as other glial tumors and because of this may be easily misdiagnosed [6–8].

The rarity of this tumor and, as a result, the limited knowledge surrounding the unique histological and radiological characteristics which differentiate this tumor type complicate our ability to obtain a prompt and accurate diagnosis. Such difficulty is furthermore complicated when a rare tumor presents outside the expected patient demographic. This was the case in the patient we present, an unusual case of a 73-year-old male with an anaplastic astroblastoma.

## 2. Case Report

### 2.1. History

A 73-year-old male presented after a fall with complaints of headaches and memory loss over the past year. The patient had a history of hypertension, hypothyroidism, and prostate cancer treated 22 years priorly. On presentation the patient was mildly confused but otherwise had no focal neurologic signs or symptoms.

### 2.2. Imaging

**Figure 1:**
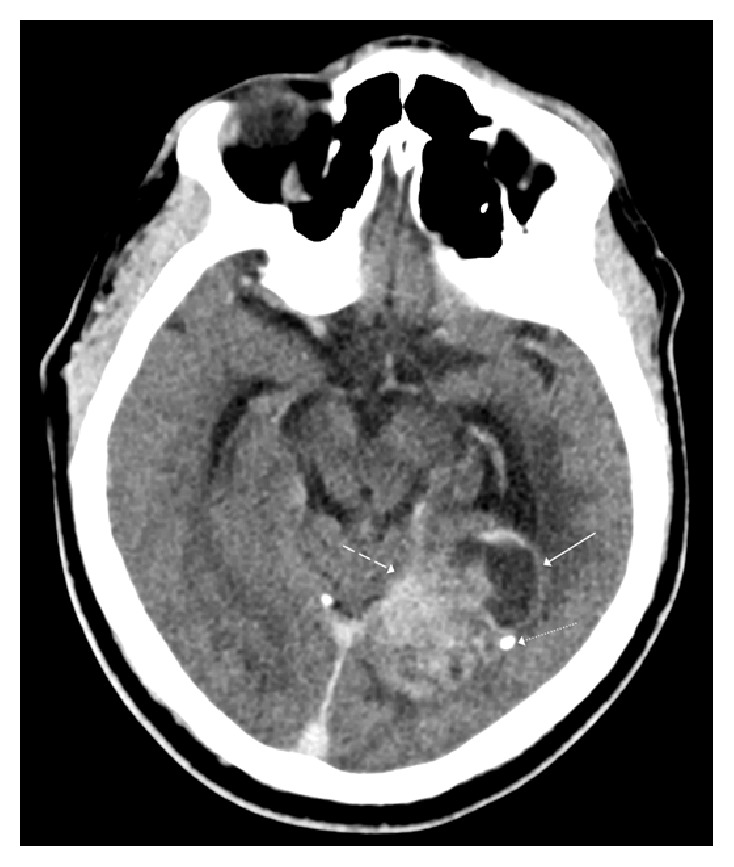
Noncontrast axial computed tomography image shows a mixed solid (dashed white arrow) and cystic (solid white arrow) temporooccipital mass with a hyperattenuating solid component and a punctate calcification peripherally (dotted white arrow).

**Figure 2:**
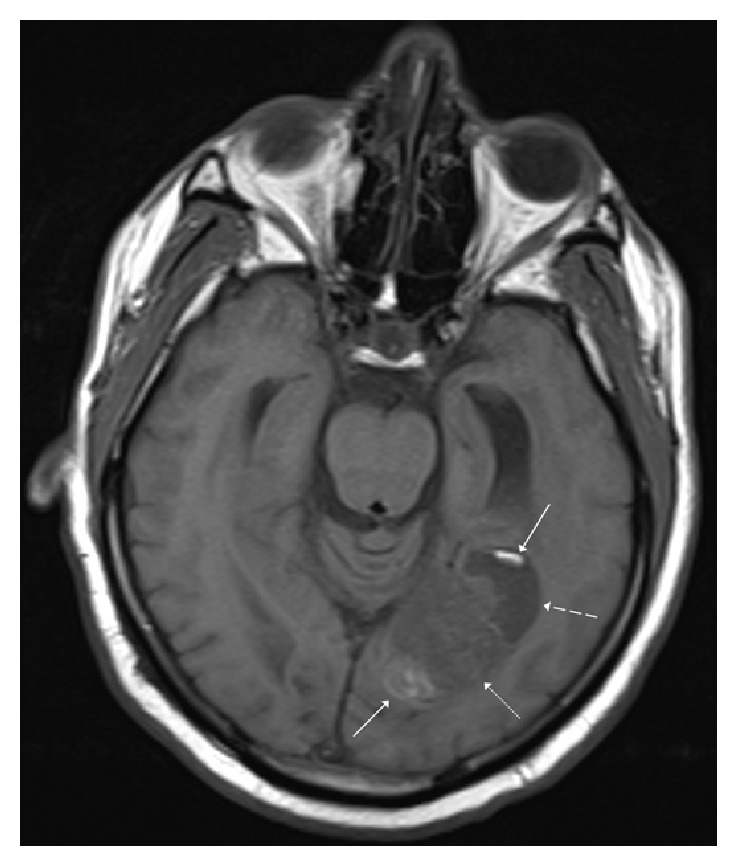
Precontrast axial T1 weighted image shows the solid component (dotted white arrows) to be hypointense to grey matter with small areas of T1 hyperintensity (solid white arrows) seen peripherally within the cystic (dashed white arrow) and solid components, likely representing areas of focal hemorrhage.

**Figure 3:**
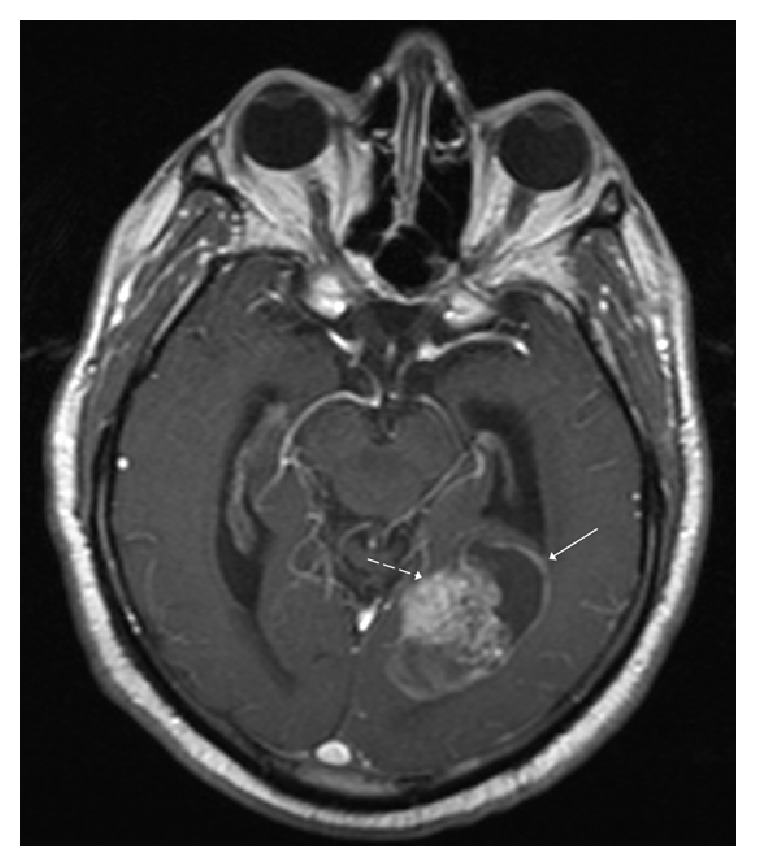
Postcontrast axial T1 weighted MR image demonstrates avid heterogeneous enhancement in the solid component (dashed white arrow) with rim enhancement of the cystic component.

**Figure 4:**
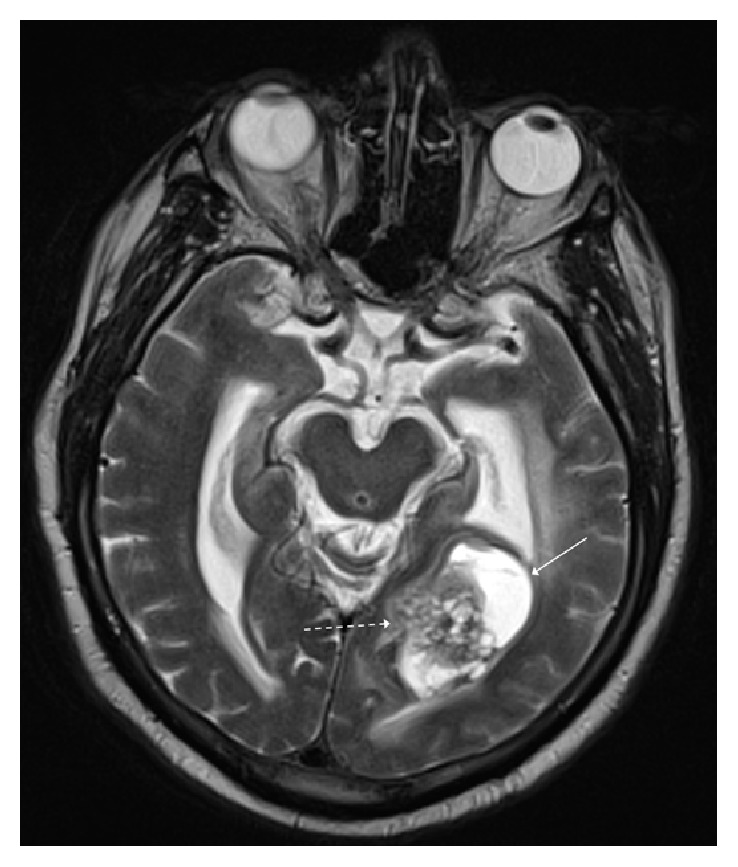
T2 weighted axial image shows a mixed solid (dashed white arrow) and cystic (solid white arrow) temporooccipital mass with a heterogeneous, bubbly appearance of the solid component.

Computed tomography (CT) of the head demonstrated a well-circumscribed partially hemorrhagic mass in the left temporal-occipital region. The mass caused effacement of the occipital horn and atrium of the left lateral ventricle as well as trapping of the temporal horn (Figure 1). MRI of the brain revealed a heterogeneously enhancing, mixed cystic and solid mass (Figures 2, 3, and 4). At this time, a differential diagnosis of glioblastoma multiforme or metastasis was proposed. A metastatic workup with CT of the chest, abdomen, and pelvis however was unremarkable. The patient and his family wanted a biopsy performed first for tissue diagnosis before they would decide on proceeding with a gross resection. A stereotactic biopsy was subsequently performed.

### 2.3. Histology

**Figure 5:**
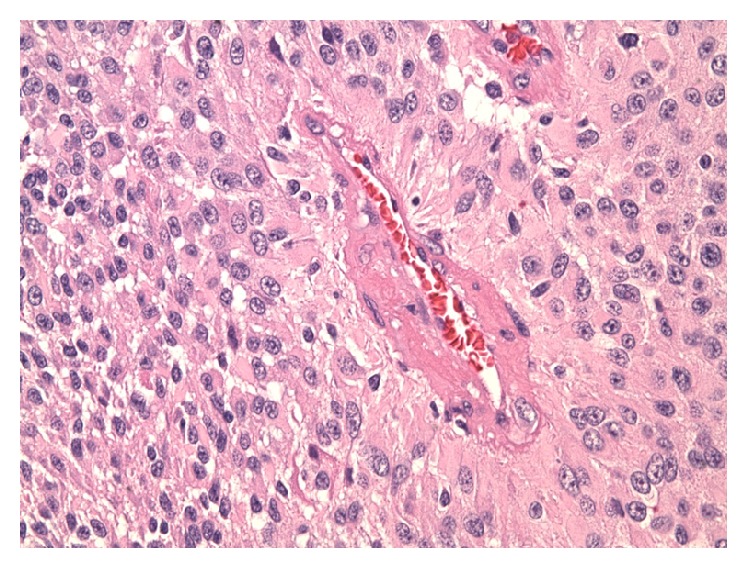
H&E stain demonstrating a perivascular pseudorosette with blunted end foot plates of the tumor cells directed toward a central blood vessel (40x).

Sections of the tumor showed a solid tumor comprised of epitheliod cuboidal-to-columnar cells with abundant eosinophilic cytoplasm and large nuclei with moderate to marked atypia. These cells demonstrated perivascular distribution in a pseudorosette pattern with broad cytoplasmic processes radiating toward the centrally placed blood vessels (Figure 5). The tumor however was nearly completely devoid of any fibrillarity. A papillary appearance was noted in multiple foci. Areas of geographic necrosis and high mitotic index of up to 11 mitotic figures per high power field were noted.

**Figure 6:**
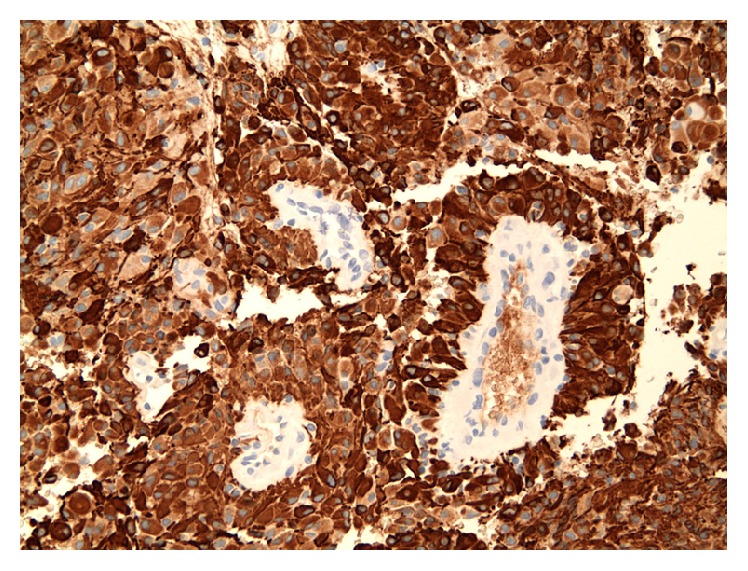
Glial fibrillary acidic protein (GFAP) stain shows positive staining demonstrating the glial origin of tumor cells (20x). Again, we can appreciate the tumor cells arranged in a perivascular pseudorosette with tumor cells directed toward the central blood vessel and the lack of fibrillarity.

Immunohistochemical stains performed showed neurofilament protein and NeuN stains to be negative within the tumor, consistent with a solid pattern of growth. The glial fibrillary acidic protein (GFAP) stain showed extensive cytoplasmic positivity (Figure 6). The CAM 5.2 immunostain was negative and epithelial membrane antigen (EMA) was expressed in a membrane and focally dot-like pattern in a subset of tumor cells. The tumor was negative for IDH (R132H) mutant protein expression. A Ki67 immunohistochemical stain showed a labeling index of 9.8%. A D2-40 immunostain showed strong cytoplasmic positivity and CD99 was extensively expressed in a membranous pattern. Patchy OLIG2 staining was also noted. These histologic and immunohistochemical findings were consistent with a diagnosis of anaplastic astroblastoma.

### 2.4. Postoperative Course

Three weeks after the initial biopsy, a left occipital craniotomy for gross total resection was performed. The tumor was cystic, rubbery, and tan-yellow in appearance and demonstrated extension into the lateral ventricle. Histological analysis again demonstrated a solid tumor comprised of epitheliod cells with abundant eosinophilic cytoplasm, large nuclei, and a lack of fibrillarity. Numerous examples of tumor cells arranged in perivascular pseudorosettes were again noted. The immunohistochemical staining pattern was consistent with that observed from the tissue obtained during the stereotactic biopsy, confirming the diagnosis of anaplastic astroblastoma. Adjuvant radiotherapy of 60 gray in 30 fractions was administered to the patient. Clinically, the patient improved, demonstrating mild confusion with an otherwise nonfocal neurological exam. Two years after the initial resection however, the patient presented with worsening mental status and was found to have recurrence of this tumor. Repeat resection was performed which again demonstrated tissue consistent with anaplastic astroblastoma. During his postoperative course his mental status continued to remain poor. He was discharged to hospice care and later expired.

## 3. Discussion

Astroblastomas are almost exclusively supratentorial; they frequently show calcification and are peripherally located. They have both solid and a multicystic component giving the distinctive bubbly appearance, characteristic of this tumor [4, 7, 9–11]. On MRI, they have relatively little peritumoral T2 hyperintensity despite their large size, suggesting a lack of tumor infiltration into local tissue [7]. Due to the relative difficulty in differentiating between astroblastomas and ependymomas on histology, it is recommended that radiologic findings demonstrating a suspicion for astroblastoma be communicated to the pathologist [12, 13]. In comparison to astroblastomas, ependymomas are frequently observed in the posterior fossa and do not commonly show the bubbly appearance characteristic of astroblastomas [11]. Radiologic imaging in the case we present was consistent with many of the features described above, such as the supratentorial location of these tumors and the characteristic solid and cystic, bubbly appearance with little surrounding T2 hyperintensity (Figure 4).

The histogenesis of anaplastic astroblastoma is controversial; however tanycytes, glial precursor cells, have been suggested as a potential tissue of origin [14–16]. The diagnosis requires a well-defined margin with the presence of perivascular pseudorosettes with thick and short, blunted tumor cells which do not taper as they project toward the central blood vessel [7, 9, 10, 17, 18]. The perivascular structures can be uniform or loosely scattered structures with round to oval nuclei and may exhibit chromatin aggregation [12]. Hyalinization and fibrotic vessel walls can be visible with occasional areas of infarcted brain tissue [10, 19]. In comparison to astroblastomas, ependymomas show some subtle but very important histological differences. The pseudorosettes of ependymomas have cell processes which taper toward the central blood vessel compared with the cell processes of astroblastomas which do not taper in this manner. True rosettes and areas of fibrillarity may be observed in ependymomas while astroblastomas are characteristically devoid of fibrillarity and do not have true rosettes [11, 13, 17]. Astroblastomas show reactivity to S-100, GFAP, and their cell membranes may be EMA reactive [2, 6, 10, 18, 20]. The Ki-67 proliferation indices range from 1% to 18%; however this does not correlate with outcome [10, 21]. A number of chromosomal aberrations have been described in small series and include gains of chromosomes 19 and 20q [10, 17, 22, 23]. There are two variants, anaplastic or high-grade astroblastoma and well differentiated or low-grade astroblastoma. The anaplastic variant displays atypical cells, more obvious mitotic activity, necrosis, and disorganized cell architecture [6, 19]. Pathological assessment of our specimen demonstrated the anaplastic variant.

Astroblastomas show a slight female predominance and are often noted in the literature to be a pediatric tumor [4, 5, 7, 24–26] with congenital lesions also reported [17, 18, 27–29]. In our review however we note many reports of this tumor presenting in adult patients and the incidence of this is well described [3–5, 7, 9, 16, 17, 24, 30–32]. Ahmed et al., for example, carried out the largest retrospective analysis that we identified in the literature and out of 239 cases, 168 were above 21 years of age. With this shift in thinking, the tumor may be considered more frequently in the differential diagnosis of adults of all ages presenting with primary brain tumors who have imaging studies characteristic for this type of tumor. Our case of an astroblastoma in a 73-year-old was uncharacteristic of this tumor type but these tumors by no means appear to be limited to a pediatric and young adult population.

The treatment of astroblastoma is not well-established owing to its rarity but surgery continues to play a vital role in the management of this condition. Complete resection is curative in low-grade cases [30, 31, 33]. In contrast to this, high-grade astroblastomas have a worse prognosis due to higher recurrence rates and more rapid progression and invasion of local brain regions [16, 19]. More aggressive treatment and close follow-up are warranted in these cases [8, 22, 30, 33, 34]. It is also suggested that the extent of peritumoral edema or peritumoral T2 hyperintensity associated with an astroblastoma on MRI may also be a feature predictive of recurrence, independent of the grade of tumor [35]. Radiotherapy has been recognized as an important adjuvant therapy in a number of high-grade astroblastoma cases [19, 29, 32], as well as following the recurrence of a low-grade lesion [30]. This differs from the treatment of ependymoma where the current standard of treatment utilizes radiotherapy in all cases, not just high-grade or recurrent cases, further highlighting the importance of accurately differentiating these two tumor types [13].

Ahmed et al. performed a retrospective analysis involving two hundred and thirty-nine patients with astroblastoma and noted a median overall survival of 55 months in patients receiving treatment. They also noted a decreased survival associated with increasing age at presentation. Though not yet proven, it is suggested that this may be associated with genetic differences in these tumors akin to the differences observed between glioblastoma cases seen in pediatric versus adult populations [5]. Though the majority of astroblastomas present in a supratentorial location, infratentorial tumors were shown to have a better prognosis [5].

## 4. Conclusion

Astroblastomas are rare, potentially curable primary brain tumors which can be difficult to diagnose. The literature often refers to this as a tumor frequently found in pediatric and young adult populations; however our patient presented with this tumor at 73 years of age. In our review we note many cases of astroblastoma reported which present in adults with some series showing a higher incidence in the adult population. The occurrence of this tumor in the elderly is also well described. We propose that this tumor is better referred to as a primary brain tumor presenting frequently in both pediatric and adult populations. This change in thinking will favor considering astroblastoma in the differential diagnosis when assessing adult patients who present with imaging findings characteristic of this rare tumor. In doing so, we may avoid any possible delays in diagnosis or misdiagnosis that might occur when overlooking this tumor as a potential primary brain neoplasm affecting adults.
